# Computed tomography features of cerebrovascular complications in intensive care unit patients with severe COVID-19

**DOI:** 10.1590/0100-3984.2021.0023

**Published:** 2021

**Authors:** Vinícius de Padua Vieira Alves, Ananda Altoé, Vanessa Veloso, Clecia Lucia Santos Ferreira, Nina Ventura, Diogo Goulart Corrêa

**Affiliations:** 1 Universidade Federal Fluminense (UFF), Niterói, RJ, Brazil.; 2 Instituto Estadual do Cérebro Paulo Niemeyer, Rio de Janeiro, RJ, Brazil.

**Keywords:** Coronavirus infections/complications, Cerebrovascular disorders, Blood coagulation disorders, Tomography, X-ray computed, Infecções por coronavírus/complicações, Transtornos cerebrovasculares, Transtornos da coagulação sanguínea, Tomografia computadorizada

## Abstract

**Objective:**

To report the computed tomography (CT) features of acute cerebrovascular complications in severely ill patients with confirmed coronavirus disease 2019 (COVID-19) in the intensive care unit.

**Materials and Methods:**

We conducted a retrospective analysis of 29 intensive care unit patients with confirmed COVID-19 who underwent CT of the brain. We describe the CT features of the cerebrovascular complications of COVID-19, as well the demographic characteristics and clinical features, together with the results of laboratory tests, such as complete blood cell count, coagulation testing, renal function testing, and C-reactive protein assay.

**Results:**

Two patients were excluded because of brain death. Among the remaining 27 patients, CT revealed acute cerebrovascular complications in six (three men and three women; 49-81 years of age), whereas no such complications were seen in 21 (15 men and six women; 36-82 years of age).

**Conclusion:**

Radiologists should be aware of the risks of cerebrovascular complications of COVID-19 and the potential underlying etiologies. COVID-19-associated coagulopathy is likely multifactorial and may increase the risk of ischemic and hemorrhagic infarction.

## Abstract (pt)

**Objetivo:**

Relatar as características da tomografia computadorizada (TC) das complicações cerebrovasculares agudas em pacientes com doença do coronavírus 2019 (COVID-19) grave, internados em unidade de terapia intensiva.

**Materiais e Métodos:**

Foi realizada análise retrospectiva de 29 pacientes com COVID-19 confirmada, internados em unidade de terapia intensiva, que realizaram TC de crânio. Descrevemos as alterações tomográficas das complicações cerebrovasculares, bem como as características demográficas, clínicas e exames laboratoriais, tais como hemograma completo, teste de coagulação, função renal e proteína C reativa desses pacientes.

**Resultados:**

Seis pacientes (três homens e três mulheres; faixa etária de 49-81 anos) apresentaram alterações cerebrovasculares reveladas por TC e 21 pacientes (15 homens e 6 mulheres; faixa etária de 36-82 anos) não apresentaram complicações cerebrovasculares agudas. Dois pacientes foram excluídos por morte encefálica.

**Conclusão:**

Os radiologistas devem estar cientes dos riscos de complicações cerebrovasculares da COVID-19 e das possíveis etiologias subjacentes. A coagulopatia relacionada à COVID-19 é provavelmente multifatorial e pode aumentar os riscos de infarto isquêmico e hemorrágico.

## INTRODUCTION

The outbreak of coronavirus disease 2019 (COVID-19), caused by infection with severe acute respiratory syndrome coronavirus 2 (SARS-CoV-2), spread rapidly to countries around the world after emerging in the city of Wuhan, China. The most common symptoms of COVID-19 are fever, cough, and fatigue. In severe cases, patients may develop pneumonia, acute respiratory distress syndrome, acute cardiac problems, and multiorgan failure^(1,2)^. Because of the neurotropic and neuroinvasive potential of SARS-CoV-2, some COVID-19 patients develop neurological manifestations, such as olfactory/taste disorders, seizures, changes in mental status, and encephalitis^(3,4)^. It is of note that coagulation disorders have also begun to be recognized as clinically relevant complications of COVID-19, some patients developing thrombosis, pulmonary embolisms, and hemorrhagic disorders. In addition, acute cerebrovascular events, specifically intracerebral hemorrhage^(5)^ and ischemic stroke^(6)^, have been observed in COVID-19 patients, even in those who are young^(6-8)^.

The objective of the present study was to report the computed tomography (CT) features of acute cerebrovascular complications in intensive care unit (ICU) patients with severe COVID-19.

## MATERIALS AND METHODS

### Subjects

This was a retrospective analysis of consecutive patients diagnosed with COVID-19 and admitted to the ICU between April 1 and May 4, 2020, with severe community-acquired pneumonia, as defined in the American Thoracic Society guidelines^(9)^. All of the patients had a definitive diagnosis of COVID-19 based on the clinical presentation and positive results from real-time reverse-transcription polymerase chain reaction nucleic acid testing of throat or nasal swab specimens.

All participants underwent CT of the brain and laboratory tests, typically including a complete blood cell count, coagulation testing, renal function testing, and C-reactive protein assay, performed according to the clinical care needs of the patients.

The Institutional Review Board of the Paulo Niemeyer Brain Institute of the State of Rio de Janeiro, Brazil, approved this retrospective observational study and waived the requirement for written informed consent. The hospital was a designated COVID-19 treatment center from April to June of 2020.

### CT protocol

Unenhanced CT scans were performed in a 16-slice scanner (Somatom Emotion 16; Siemens Healthineers, Erlangen, Germany) with the following parameters: rotation time, 1 s; detector collimation, 0.6 mm; voltage, 130 kV; current, 200 mA; slice thickness, 2 mm; and pitch, 1.0. All CT results were evaluated for signs of recent cerebrovascular pathology, such as brain hemorrhages and acute or subacute ischemic alterations, by two radiologists with 10 and 2 years of experience, respectively.

### Statistical analysis

Demographic variables (age and sex distribution) and clinical variables (leukocyte, lymphocyte, and neutrophil counts, as well as serum levels of urea, creatinine, and C-reactive protein) were compared between the COVID-19 patients with recent cerebrovascular complications observed on CT (w/CVC group) and those without (w/oCVC group). Mann-Whitney tests or Fisher’s exact tests (for categorical variables) were used as appropriate. All statistical analyses were performed with the IBM SPSS Statistics software package, version 20.0 (IBM Corp., Armonk, NY, USA).

## RESULTS

During the study period, 29 consecutive patients were admitted to the ICU with confirmed SARS-CoV-2 infection and underwent CT of the chest and brain on the same day. Of those 29 patients, 21 (15 men and six women; age range, 36-82 years) were in the w/oCVC group and six (three men and three women; age range, 49-81 years) were in the w/CVC group. The two remaining patients presented with diffuse cerebral edema visible on brain CT scans and ultimately evolved to clinically confirmed brain death, therefore being excluded from the analysis. The patients in both groups were receiving prophylactic anticoagulation in accordance with our ICU protocol for patients with COVID-19. The sociodemographic and clinical characteristics of the six patients in the w/CVC group (designated patients 1-6, respectively) are summarized in Table 1.

**Table 1:** Clinical characteristics of six patients with severe COVID-19 and cerebrovascular complications.

Characteristic	Patient 1	Patient 2	Patient 3	Patient 4	Patient 5	Patient 6
Age	80	81	56	56	49	61
Sex	Female	Male	Male	Female	Male	Female
Comorbidities	Arterial hypertension, diabetes mellitus, heart disease, atrial fibrillation, and previous ischemic stroke	None	None	Arterial hypertension and diabetes mellitus	None	Arterial hypertension and obesity
Mechanical ventilation required	Yes	Yes	Yes	Yes	Yes	Yes

**Figure 1.**
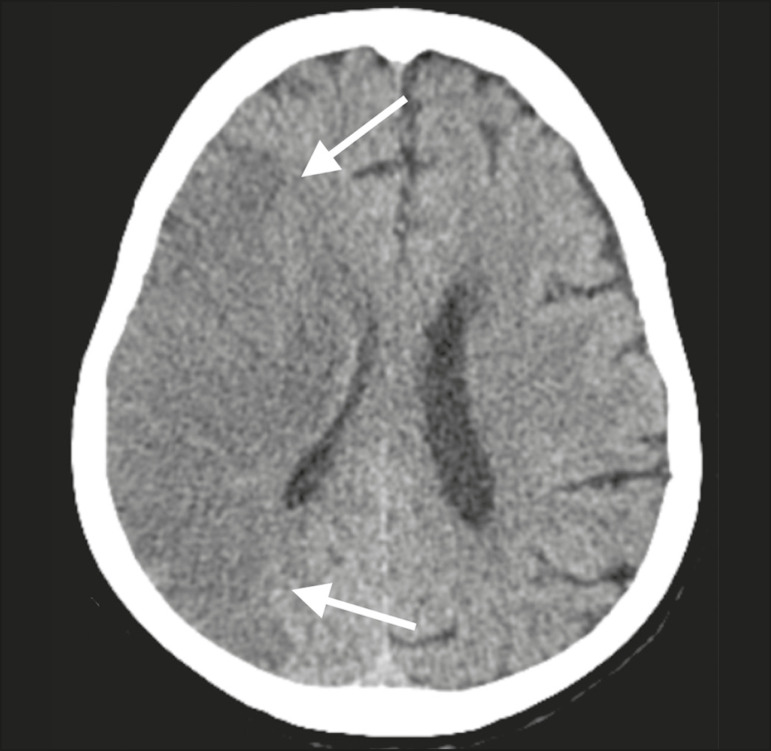
Case 1. Brain CT of an 80-year-old woman with COVID-19 showing an extensive acute ischemic stroke, affecting the right MCA territory (arrows).

**Figure 2.**
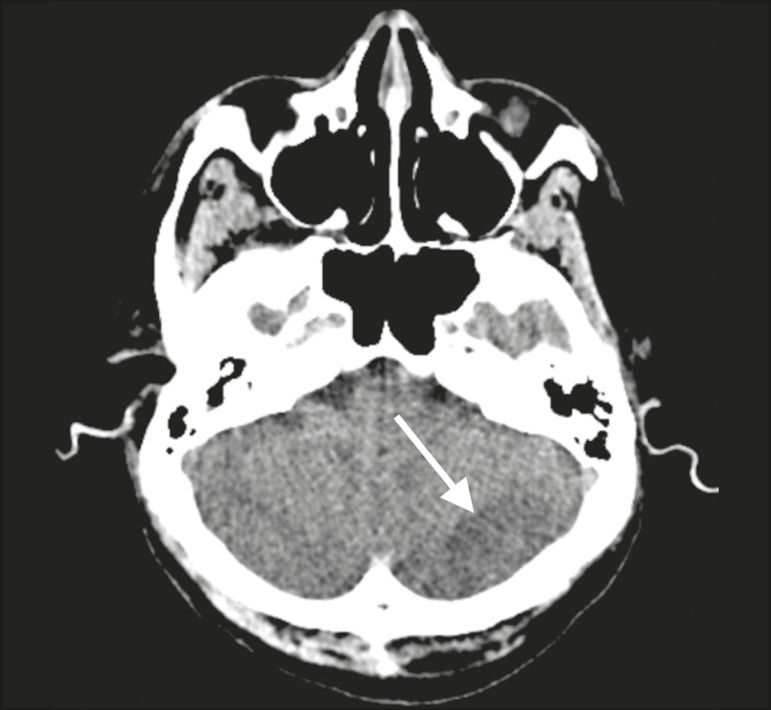
Case 2. Brain CT of an 81-year-old man with COVID-19 showing an acute ischemic stroke in the left cerebellar hemisphere, in the posterior inferior cerebellar artery territory (arrow).

**Figure 3.**
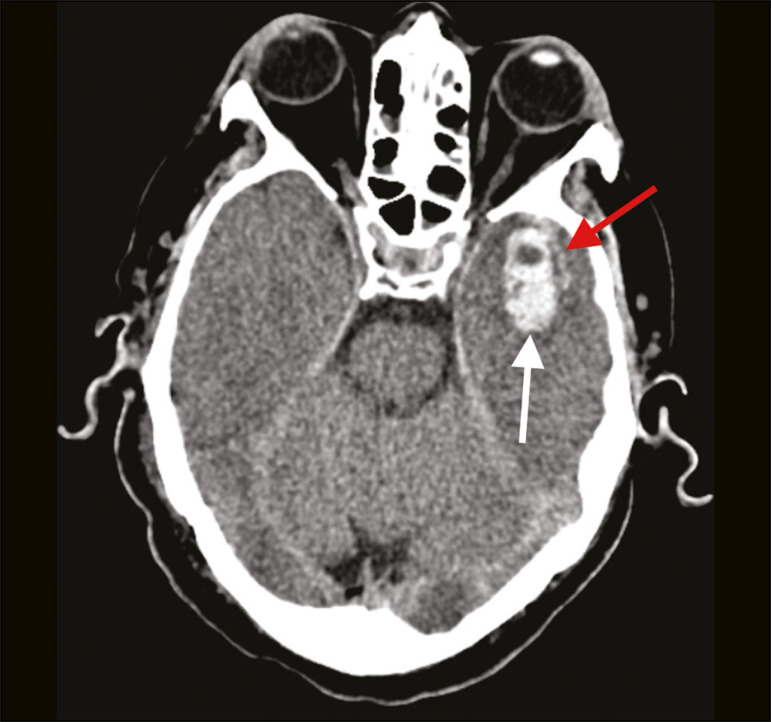
Case 3. Brain CT of a 56-year-old man with COVID-19 showing an intraparenchymal hematoma in the anterior pole of the left temporal lobe (white arrow), together with subarachnoid hemorrhage (red arrow). The patient also had an acute ischemic stroke in the left frontal lobe (not shown).

**Figure 4.**
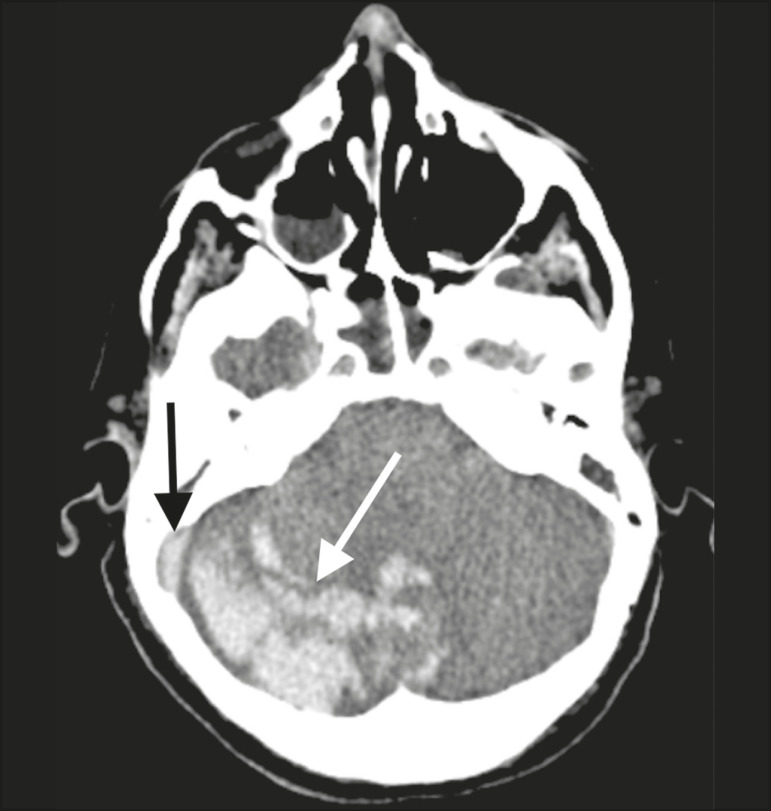
Case 4. Brain CT of a 56-year-old woman with COVID-19 showing a hematoma in the vermis and right cerebellar hemisphere (white arrow), together with a hyperdense area throughout the ipsilateral sigmoid and transverse sinuses (black arrow) suggestive of dural sinus thrombosis as the cause of the cerebellar hematoma.

**Figure 5.**
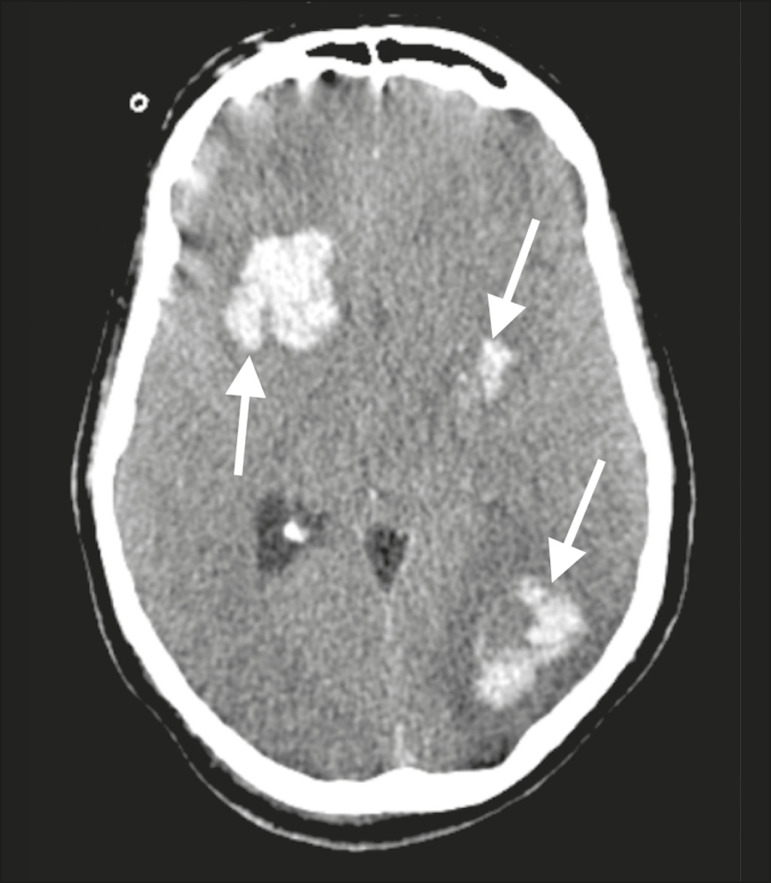
Case 5. Brain CT of a 49-year-old man with COVID-19 showing multiple lobar and basal ganglia hematomas (arrows).

**Figure 6.**
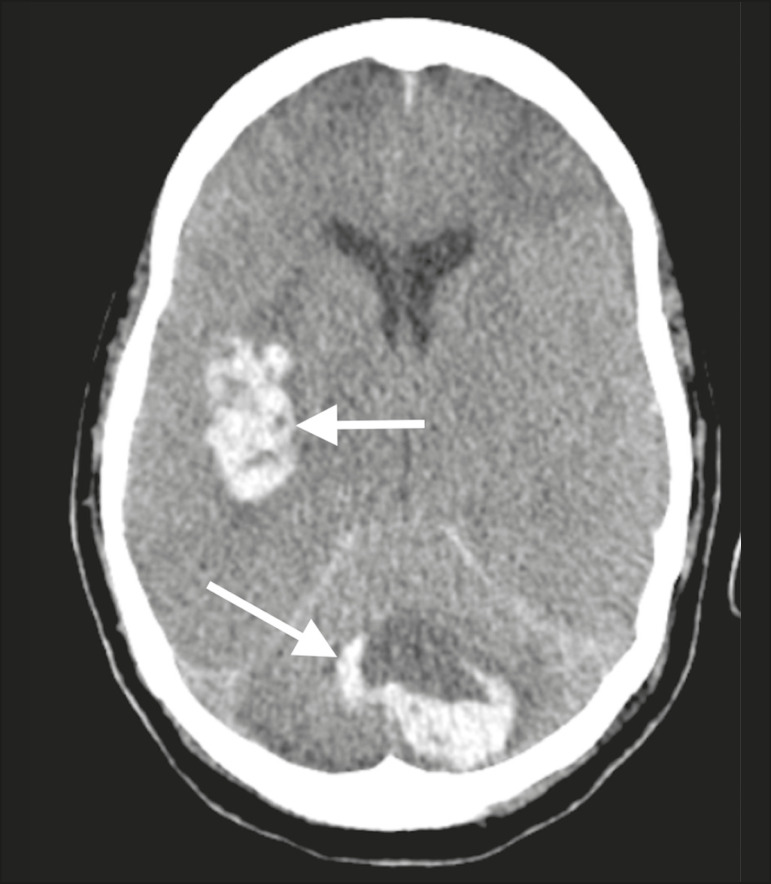
Case 6. Brain CT of a 61-year-old woman with COVID-19 showing supratentorial and infratentorial hematomas (arrows). The patient also had acute ischemic strokes in the left parieto-occipital and right parietal lobes, with hemorrhagic transformation (not shown).

Patient 1 presented with an extensive acute ischemic stroke affecting the right middle cerebral artery (MCA) territory that was accompanied by a hyperdense artery (Figure 1). Patient 2 had an acute ischemic stroke in the left cerebellar hemisphere in the territory of the posterior inferior cerebellar artery (Figure 2). Patient 3 had an acute ischemic stroke in the left frontal lobe, together with an intraparenchymal hematoma in the anterior pole of the left temporal lobe adjacent to a subarachnoid hemorrhage (Figure 3). Patient 4 had a hematoma in the vermis and right cerebellar hemisphere accompanied by a hyperdense area encompassing the ipsilateral sigmoid and transverse venous sinus, suggestive of dural sinus thrombosis (Figure 4). Patient 5 had multiple lobar and basal ganglia hematomas (Figure 5). Patient 6 presented with acute ischemic strokes in the left parieto-occipital and right parietal lobes, with hemorrhagic transformation, together with multiple supratentorial and infratentorial hematomas (Figure 6).

All six of the patients in the w/CVC group required tracheal intubation and mechanical ventilation, compared with only seven of the 14 patients in the w/oCVC group, although the difference was not statistically significant (Table 2). There was a borderline significant difference between the two groups in terms of the leukocyte counts, which were higher in the w/CVC group. The two groups were statistically similar with respect to sex, age, neutrophil counts, lymphocyte counts, and platelet counts, as well as serum levels of C-reactive protein, urea, and creatinine. Activated partial thromboplastin time, prothrombin time, and the international normalized ratio did not differ significantly between the two groups.

**Table 2:** Comparative analysis of COVID-19 patients with and without acute cerebrovascular complications.

Variable	Group		*P*
	w/CVC (n = 6)	w/oCVC (n = 21)	
Age (years), mean ± SD	63.83 ± 13.46	58.85 ± 12.53	0.48*
Sex, n (%)			
Male	3 (50.0)	15 (71.4)	0.36^†^
Female	3 (50.0)	6 (28.6)	
Leukocytes (cells/µL), mean ± SD	22116.66 ± 7242.87	15328.57 ± 6578.12	0.04*
Neutrophils (cells/µL), mean ± SD	15880.00 ± 4234.61	11649.19 ± 5429.54	0.06*
Lymphocytes (cells/µL), mean ± SD	2413.33 ± 1421.23	1542.23 ± 631.09	0.22*
Platelets (cells/µL), mean ± SD	224166.70 ± 154971.7	267142.90 ± 125903.6	0.19*
C-reactive protein (mg/L), mean ± SD	326.55 ± 242.55	213.83 ± 145.59	0.85*
Urea (mg/dL), mean ± SD	104.00 ± 40.76	63.14 ± 39.54	0.06*
Creatinine (mg/dL), mean ± SD	3.32 ± 1.77	1.71 ± 1.46	0.34*
Activated partial thromboplastin time (s), mean ± SD	27.51 ± 7.016	27.24 ± 4.91	0.92*
Prothrombin time (s), mean ± SD	19.70 ± 6.08	16.34 ± 3.85	0.26*
International normalized ratio, mean ± SD	1.55 ± 0.48	1.28 ± 0.31	0.26*
Mechanical ventilation required, n (%)			
Yes	6 (100.0)	14 (66.7)	0.15^†^
No	0 (0.0)	7 (33.3)	

* Mann-Whitney test;

† Fisher's exact test.

## DISCUSSION

In the present study, we present six critical cases of COVID-19 with cerebrovascular complications characterized by ischemic and hemorrhagic features. Two additional patients presented with diffuse cerebral edema, related to brain death.

COVID-19 has been the subject of a series of recent publications in the radiology literature of Brazil^(10-14)^. Because COVID-19 is not limited to respiratory manifestations, radiologists should be aware of the heightened risk of cerebrovascular events in this population and their potential underlying etiologies^(15,16)^. COVID-19-associated cerebrovascular disease appears to involve ischemic and hemorrhagic coagulation disturbances secondary to endothelial damage due to direct viral invasion^(17)^. SARS-CoV-2 can enter cells through angiotensin-converting enzyme 2 (ACE2) receptors^(15,17)^, generating an exacerbated inflammatory reaction, characterized by macrophage proliferation and hypersecretion of cytokines, which leads to vascular damage that may increase the risk of ischemic and hemorrhagic infarction^(16)^. 

Expression of ACE2 occurs throughout the vascular endothelial tissues and in the brain^(15,18,19)^. That distribution may explain some major features of COVID-19-associated cerebrovascular disease and might contribute to hemorrhagic complications^(19)^. However, the increased predisposition of patients with COVID-19 to develop acute cerebrovascular complications is multifactorial. The binding of SARS-CoV-2 to ACE2 may lead to endothelial damage and increased subintimal inflammation, which are followed by hemorrhage, with or without thrombosis. The virus can also increase the expression and activity of ACE2, resulting in renin-angiotensin system imbalance, which is associated with increased atherosclerosis^(20)^. In addition, hyperinflammation with hyperferritinemia and high D-dimer values can lead to a hypercoagulability state^(21,22)^, with an increased risk of ischemic events^(15,22,23)^. Furthermore, the acute severe inflammatory response to SARS-CoV-2 results in decreased levels of circulating lymphocytes, together with a secondary shift of the immune defense system toward natural killer cells, circulating macrophages, and neutrophils, as well as significantly elevated levels of proinflammatory cytokines and chemokines-including interleukin (IL)-2, IL-6, IL-7, IL-10, IL-1β, IL-18, interferon-γ, tumor necrosis factor-α, granulocyte colony-stimulating factor, monocyte chemoattractant protein-1, and macrophage inflammatory protein 1-α-all of which contribute to various processes in cerebrovascular ischemia^(24,25)^. Antiphospholipid antibodies are also detected in the plasma of patients with severe COVID-19, those antibodies aggravating the hypercoagulability state through the formation of immune complexes. Finally, hyperglycemia in COVID-19 patients may trigger cerebrovascular disease by increasing oxidative stress and blood viscosity^(20,24)^.

Parenchymal hematoma, subarachnoid hemorrhage, and subdural hematoma have all been associated with COVID-19. In general, patients with intracranial hemorrhages associated with COVID-19 are younger than are patients with intracranial hemorrhages due to other causes, have no history of vascular abnormality, and suffer from lobar parenchymal hematoma, which can be multifocal. However, it remains unknown whether COVID-19 has a causal relationship with intracranial hemorrhages through ACE2 inactivation, endothelial dysfunction/degeneration, coagulopathy, or hypocoagulability, or rather, whether secondary effects of COVID-19, such as renal failure, and concomitant therapeutic anticoagulation in a critically ill population is the culprit. The atypical, multifocal nature of many of the reported brain hemorrhages to date would suggest some form of underlying vasculopathy which likely acts synergistically with the previously mentioned factors in causing intracranial hemorrhages^(26)^. In the present study, we describe four cases of intracranial hemorrhage-two in patients with no known risk factors and two in patients with multilobar hematomas-corroborating the hypothesis that the bleeding is associated with a form of vasculopathy related to the infection.

In our case series, despite undergoing anti-thrombotic therapy, one patient presented a hyperdense MCA due to an arterial thrombus and another patient had a hyperdense thrombus in the right transverse and sigmoid sinuses, together with a hemorrhagic venous infarction. Although the full details of COVID-19-induced pathophysiology remain to be elucidated, several cerebrovascular risk factors have been associated with severe COVID-19, including cardiovascular disease, diabetes mellitus, arterial hypertension, smoking, advanced age, and history of stroke. That raises the question of whether their relationship is causal or if they just coincide^(26)^. It is possible that cerebrovascular complications occur more frequently in severely and critically ill patients. In our patient sample, it was not possible to delineate clinical risk factors for cerebrovascular complications, because all of the patients had severe pneumonia, and only one of the clinical parameters analyzed differed significantly between the w/CVC and w/oCVC groups.

Our findings extend the available literature regarding cerebrovascular disease associated with severe or critical COVID-19. Notably, Mao et al.^(3)^ reported a 5.7% prevalence of acute cerebrovascular disease in a sample of 88 patients with severe COVID-19 and Helms et al.^(23)^ reported a 4.6% prevalence of acute/subacute ischemic stroke in a sample of 64 ICU patients with COVID-19. In addition, Oxley et al.^(8)^ reported five cases of large-vessel stroke in COVID-19 patients under 50 years of age in an emergency department setting. Similarly, Kandemirli et al.^(27)^ reported one case of acute transverse sinus thrombosis and another case of acute stroke affecting the right MCA territory in a sample of 27 ICU patients with COVID-19. Four other cases of deep cerebral vein thrombosis in patients with COVID-19 have been reported, including two with hemorrhagic venous infarctions^(28,29)^. The relatively high proportion of patients with cerebrovascular complications in our sample, compared with those of previous studies, can be attributed to the older age and ICU selection bias of our sample, in which, accordingly, the prevalence of comorbidities was higher.

Although intravenous thrombolysis is not contraindicated in patients with COVID-19, leukocytosis, an elevated C-reactive protein level, and an elevated D-dimer level are risk factors for hemorrhagic transformation, as well as for post-thrombolysis mortality^(30,31)^. Some authors have found that the risk of hemorrhage is higher among patients with COVID-19-related stroke who receive intravenous thrombolysis than among those who do not^(32)^. However, another study demonstrated that intravenous thrombolysis may be safe in COVID-19 patients^(33)^. At the moment, there is no clear evidence that the use of intravenous thrombolysis is contraindicated in COVID-19 patients with abnormal biochemical indicators^(20)^. None of the patients in our sample were treated with venous thrombolysis, and some underwent hemorrhagic transformation regardless of treatment, showing the predisposition to hemorrhage in these patients.

Our study has some limitations. Because it was a retrospective, observational case series, it was not possible to establish casual relationships. In addition, we conducted only unenhanced CT examinations without angiography, in accordance with the clinical decisions of the attending ICU physicians. Finally, the power of our statistical analysis was limited by the relatively small group sizes. Despite these limitations, it was possible to evaluate and document cerebrovascular complications in critically ill patients with COVID-19.

In conclusion, patients with COVID-19 requiring ICU support are at risk of developing acute cerebrovascular complications, which may be related to coagulation disorders or previous comorbidities. Radiologists should be attentive to signs of cerebrovascular complications as a possible cause of clinical worsening, beyond pulmonary infection, and should consider imaging studies to rule out neurological complications.
